# *DPYD* Exon 4 Deletion Associated with Fluoropyrimidine Toxicity and Importance of Copy Number Variation

**DOI:** 10.3390/curroncol30010051

**Published:** 2023-01-04

**Authors:** Theodore J. Wigle, Samantha Medwid, Cameron Ross, Ute I. Schwarz, Richard B. Kim

**Affiliations:** 1 Department of Physiology & Pharmacology, Western University, London, ON N6A 3K7, Canada; 2 Department of Medicine, Western University, London, ON N6A 3K7, Canada

**Keywords:** fluoropyrimidines, dihydropyrimidine dehydrogenase deficiency, adverse drug reactions, pharmacogenetics, copy number variation

## Abstract

Fluoropyrimidine chemotherapy is associated with interpatient variability in toxicity. A major contributor to unpredictable and severe toxicity relates to single nucleotide variation (SNV) in dihydropyrimidine dehydrogenase (*DPYD*), the rate-limiting fluoropyrimidine metabolizing enzyme. In addition to SNVs, a study of Finnish patients suggested that a *DPYD* exon 4 deletion was observed in their population. To better understand the potential generalizability of such findings, we investigated the presence of this exon 4 deletion in our Canadian patient population, using a TaqMan assay. We selected 125 patients who experienced severe fluoropyrimidine-associated toxicity, and 125 matched controls. One patient in the severe toxicity group harbored a haploid *DPYD* exon 4 deletion, and required a 35% dose reduction after their first fluoropyrimidine treatment cycle due to toxicity and required an additional 30% dose reduction before tolerating treatment. The predicted allele frequency was 0.2% in our cohort, much lower than the 2.4% previously reported. We also carried out a literature review of copy number variation (CNV) in the *DPYD* gene, beyond fluoropyrimidine toxicity and show that various types of CNV in *DPYD* are present in the population. Taken together, our findings suggest that CNV in *DPYD* may be an underappreciated determinant of *DPYD*-mediated fluoropyrimidine toxicity.

## 1. Introduction

Fluoropyrimidines, 5-fluorouracil (5-FU) or the prodrug capecitabine, are widely prescribed for the treatment of solid tumors [1]. Approximately 30% of patients experience severe toxicity during fluoropyrimidine chemotherapy [2]. Dihydropyrimidine dehydrogenase (DPD, gene name *DPYD*) is the rate-limiting enzyme for fluoropyrimidine catabolism [3]. Patients with DPD deficiency have reduced clearance of fluoropyrimidines and are thereby at a higher risk for toxicity [4]. Regulatory agencies, such as the Federal Drug Administration (FDA), have included drug label warnings describing this association without providing guidance on how to reduce this risk. In contrast, multiple European countries have recently published guidelines recommending pre-treatment DPD deficiency screening [5,6]. Furthermore, the European Medicines Agency also released a statement in support of DPD deficiency screening by either phenotyping or genotype testing methods prior to the use of fluoropyrimidines [7].

*DPYD* is a large pharmacogene spanning over 840 kb in length, with only 3078 bp of coding sequence [8,9]. It contains 23 relatively small exons (69–961 bp), surrounded by large intronic regions [8,9]. The *DPYD* locus harbors many single nucleotide variants (SNVs), however, only a small number of these have been confirmed to reduce enzyme activity and increase the risk of fluoropyrimidine-associated toxicity [10,11,12]. Currently, four *DPYD* SNVs are considered to be clinically relevant, *DPYD* c.1905 + 1G > A (*2A, rs3918290), *DPYD* c.1679T > G (*13, rs55886062), *DPYD* c.2846A > T (rs67376798), and *DPYD* c.1129–5923C > G (rs75017182, the causative variant of Haplotype B3, in linkage disequilibrium with *DPYD* c.1236G > A [rs56038477]). The Clinical Pharmacogenetics Implementation Consortium (CPIC) and the Dutch Pharmacogenomics Working Group (DPWG) have published guidelines advising on fluoropyrimidine dose adjustments in patients harboring one of these *DPYD* variants [6,13]. 

Despite preemptive genotype-guided dosing, 23% and 31% of *DPYD* wild-type patients in a Dutch population [14] and in our previously reported Canadian population [15], respectively, still experienced severe fluoropyrimidine-related adverse events (AEs). Previous studies have attempted to identify additional variables to explain this toxicity, such as uncharacterized *DPYD* SNVs [16]. However, SNVs are not the sole source of genetic variation within the *DPYD* locus. Other types of genetic variation such as gene copy number variation (CNV) including exon deletions have also been reported [17,18,19,20,21,22,23,24]. Deletion of the *DPYD* gene, whole or in part, results in loss of DPD enzyme activity which has been associated with intellectual disability, seizures and delayed motor skill development linked to congenital thymine-uraciluria [18,19,20,21,22,23,24,25,26,27]. Additionally, exon deletions in *DPYD* may be more common than previously thought, with it being suggested that 7% of patients with DPD deficiency may be due to a deletion in *DPYD* [28]. 

Recently, Saarenheimo et al. described a novel genomic *DPYD* exon 4 deletion in 4 of 167 Finnish patients who had undergone pre-treatment *DPYD* screening [17]. Loss of *DPYD* exon 4 is predicted to result in a truncated DPD protein, with loss of enzyme activity [17,29]. If this novel genomic exon 4 deletion is present in other populations beyond those of Finnish descent with a similar frequency, it would clearly merit consideration as a clinically actionable *DPYD* CNV for inclusion as a part of standard *DPYD* deficiency testing panel. Accordingly, we sought to screen for the presence of this genomic exon 4 deletion in our Canadian population to determine its frequency and any association with fluoropyrimidine-associated AEs during chemotherapy.

## 2. Methods

### 2.1. Patient Cohort

A cohort of 250 patients were included for this study from a previously published dataset [15]. The study was approved by the Institutional Review Board at Western University and all patients provided written informed consent. Patients with a primary adenocarcinoma of the colon or rectum were selected, with an equal proportion of men and women. All patients carrying known clinically relevant *DPYD* variants (*DPYD* c.1905 + 1G > A, *DPYD* c.1679T > G, *DPYD* c.2846A > T, or *DPYD* c.1236G > A) were excluded. Next, we defined patients with adverse events (AE) as experiencing a severe (≥Grade 3) fluoropyrimidine-associated toxicity as determined by National Cancer Institutes’ Common Terminology Criteria for Adverse Events version (CTCAE) 5.0. Patients who received fluoropyrimidine therapy without any severe toxicity-related event were deemed as controls. AE (*n* = 125) and control (*n* = 125) cohorts were matched for age, sex, and treatment regimen. 

### 2.2. Detection of DPYD Exon 4 Deletion

Whole blood samples were collected, and DNA was extracted using a MagNA pure compact instrument (Roche, Mississauga, ON, Canada). A TaqMan copy number variation (CNV) assay was used to determine the presence of a *DPYD* exon 4 deletion. We utilized a FAM-labeled probe against *DPYD* exon 4 (Thermo Fisher Scientific, Cat: Hs03083443, Waltham, MA, USA), and a Vic-labeled probe against the control gene RNAse P (Thermo Fisher Scientific, Cat: 4316844). Patients were tested on 96 well plates in batches of 30 with healthy volunteer DNA samples used as a cross-reference between plates. Relative quantification (RQ) was determined according to manufacturer instructions, and an RQ of approximately 1 was interpreted as diploid, while a 50% reduction in RQ was interpreted as a haploid deletion.

### 2.3. Literature Review for DPYD CNV

A review with focus on *DPYD* CNV was performed using MEDLINE (PubMed). Search terms included, “(*DPYD*) OR (dihydropyrimidine dehydrogenase) AND (deletion) OR (duplication) OR (copy number variation)” and “(1p21.3) AND (deletion) OR (duplication)” for articles published in English prior to 25 November 2022. Only articles that found a partial or whole genomic germline deletion or duplication in *DPYD* that resulted in an observed phenotype were included. Articles were then searched for any additional references that contained *DPYD* CNV.

## 3. Results

### 3.1. Study Population

Among 250 patients, 124 (49.6%) were male, the mean age was 65 and 238 (95%) were Caucasian (95%). There were no significant differences in fluoropyrimidine treatment characteristics (dose intensity and cycle number) or regimens between groups. Baseline demographics are reported in Table 1. 

### 3.2. Fluoropyrimidine-Associated Toxicity

There was a total of 157 toxicity events in 125 patients with adverse events, as each patient had at least one event, therefore, there are more events than patients (Table 2). The largest category was gastrointestinal events accounting for 43.3% of total events, followed by myelosuppression, with neutropenia being the most common in this category. Additionally, there were 25 cases of hand-foot syndrome (HFS, also known as palmar-plantar erythrodysesthesia). Finally, 15 events were determined to be associated with fluoropyrimidines but did not fall under the classic toxicity categories. During the study period, 5 patients in the adverse event group died due to fluoropyrimidine-associated toxicity. 

### 3.3. Exon 4 Deletion

**Figure 1:**
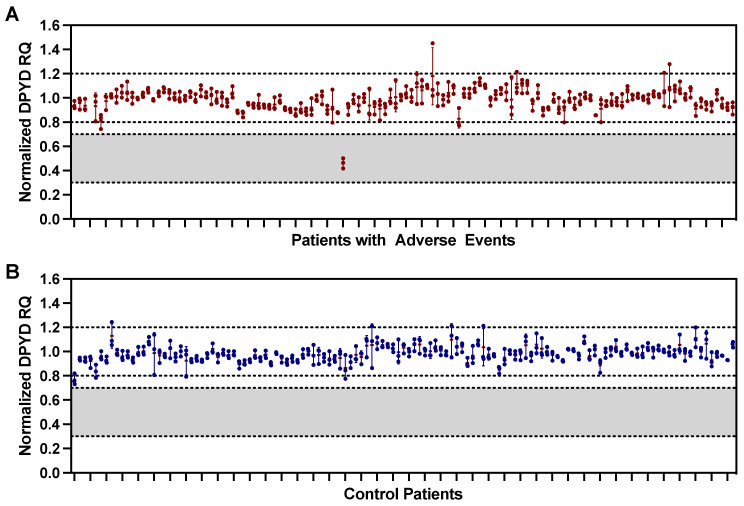
*DPYD* Exon 4 Copy Number Variation. *DPYD* exon 4 amplification relative to *RNaseP* as relative quantification (RQ) values for (**A**) adverse event and (**B**) control patients. An RQ value of 1 indicates a diploid copy number, while a ratio of 0.5 implies a haploid deletion. Points are technical replicates, bars are mean ± standard deviation.

The presence of a *DPYD* exon 4 deletion was examined in patients with adverse events (Figure 1A) and control (Figure 1B) patients. All control patients were found to be wildtype, while we detected a single patient with a haploid *DPYD* exon 4 deletion in the adverse event group (Figure 1). This was an elderly patient with stage IV colorectal adenocarcinoma. The patient received capecitabine monotherapy with palliative intent, initiated at 100% ideal dose. They then experienced a grade 2 oral mucositis during the first cycle of therapy. The capecitabine dose was reduced by 35%, however, by the end of the second cycle, they had developed grade 3 diarrhea and grade 2 HFS. Following the resolution of these adverse events, capecitabine therapy was reinitiated with an additional 30% reduction (now at 35% of ideal dose). The patient continued on capecitabine monotherapy for an additional 9 cycles before discontinuing due to a change in the goals of care. This single haploid deletion amongst 250 patients represents a frequency of 0.002, in our cohort of mostly (95%) Caucasian individuals. 

### 3.4. Literature Review of DPYD Copy Number Variation

CNV in *DPYD* was defined as genomic deletions or duplications over 1 kb that caused an observable phenotype [28,30]. In total, 20 independent CNVs in *DPYD* were discovered, of which 18 were heterozygous deletions, and two were a duplication (Table 3). Heterozygous *DPYD* exon deletions were found in 3 cases, ranging in size from 10 kb to 122 kb, resulting in deletions in exons 6, 12 and 14–16 [18,19]. While whole *DPYD* deletions ranged in size from 1.1 Mb to 14 Mb [19,20,21,23,24,25,26,27], *DPYD* duplications were 3.56–3.68 Mb in length [22]. All partial and whole deletions, as well as the *DPYD* duplications were described to result in a similar phenotype which included intellectual disability, autism-like symptoms, speech delays, and often seizures and obesity (Table 3). Of note, the degree of severity of intellectual disability and other symptoms ranged greatly between patients, from mild to severe. 

## 4. Discussion

Currently, genotype-guided testing for *DPYD* c.1905 + 1G > A, c.1679T > G, c.2846A > T, and c.1236G > A only account for 30% of toxicity seen in patients taking fluoropyrimidines [13,15]. Given the recent report suggesting a *DPYD* exon 4 deletion may be relatively common [17], we screened for this deletion in our cohort of patients who had experienced fluoropyrimidine-associated toxicity, but did not carry any of the currently clinically actionable *DPYD* SNVs [15]. In our cohort of predominantly Caucasian patient population, we only identified a single patient carrying a haploid deletion of *DPYD* exon 4 out of 250 patients (0.2%). However, this patient experienced severe fluoropyrimidine-associated toxicity and required a greatly reduced dose. 

Although the impact of single nucleotide variation (SNV) or polymorphism (SNP) is widely accepted and often considered the only major genetic determinant of drug metabolism and response, we are now increasingly aware of the role of gene deficiency or duplication, more broadly termed copy number variation (CNV), to drug toxicity or lack of efficacy [28,30]. However, the study of CNV in pharmacogenes is lacking compared to the extensive research into SNV [28]. *CYP2D6* is the most commonly studied pharmacogene in relation to CNV, where both deletions and duplications have been associated with drug response and efficacy [30]. However, Santos et al. found that 201 out of 208 pharmacogenes investigated contained novel exonic deletions or duplications, with 2611 deletions and 2978 duplications being discovered [28]. Furthermore, they reported that in 42% of the genes studied, deletions accounted for >5% of the loss of function alleles and that in the African population, *DPYD* deletions account for over 5% of the loss of function *DPYD* alleles [28]. Interestingly, *DPYD* is found to contain a common fragile site, *FRA1E*, which encompasses nearly 370 kb of the approximately 840 kb *DPYD* gene [31]. Specifically, exons 13–16 lie within the highest fragility region that accounts for 86% of all known breaks [31]. Thus, the presence of *FRA1E* may be a contributor to the extent of observed CNVs in *DPYD*.

Loss of DPD enzyme activity has been studied for many decades, particularly in relation to the accumulation of thymine and uracil, resulting in thymine-uraciluria [32,33,34,35,36]. There is wide variability in terms of clinical phenotype, but DPD deficiency has been linked to intellectual disability and autism-like symptoms and associated with partial or whole *DPYD* gene deletion as summarized in Table 3 [18,19,20,21,23,24,25,26,27]. Heterozygous deletions in *DPYD* have been associated with an intellectual disorder, including a 13.8 kb deletion (c.1340–3473_c.1525 + 10,154del13,812) resulting in a shortened transcript lacking exon 12 and a 122 kb deletion of exons 14 and 15 (c.1741_2058del) [18]. Other heterozygous microdeletions of 1p21.3 which contains the whole *DPYD* gene, as well as MIR137, have been associated with intellectual disability and autistic spectrum disorder [19,21]. Furthermore, a gene duplication in the same region, 1p21.3p21.2 has been reported, interestingly, this patient had very similar symptoms to patients with a deletion in this region [22]. Whether these symptoms are due to a loss of *DPYD*, MIR137 or other genes in the region remains unknown. Furthermore, it was reported that in DPD-deficient patients, genomic deletions in *DPYD* account for 7% of cases and that the frequency of DPYD deletions may be ethnicity-specific [18,28]. Additional CNV in *DPYD* can also be found in the Database of Genomic Variants (http://dgv.tcag.ca/dgv/app/home; accessed on 28 November 2022) and DatabasE of genomiC varIation and Phenotype in Humans using Ensembl Resources (DECIPHER, https://www.deciphergenomics.org/; accessed on 28 November 2022). 

Nevertheless, the association of *DPYD* CNVs with fluoropyrimidine toxicity is less clear. A small study that investigated *DPYD* CNVs found no deletions or duplications in 68 patients with severe fluoropyrimidine-associated toxicity [37]. Additionally, a study of 234 Spanish patients treated with fluoropyrimidines found no deletions or duplications in *DPYD* [38]. Another study reported a variant at the *DPYD* splice donor site c.321 + G > A causing the deletion of exon 4 (rs746368304) [29]. The mature mRNA was lacking an 88 bp section (r.234_321), resulting in a loss of amino acids 78–107 [29]. Deletion of exon 4 creates a premature stop codon, p.Cys79Thrfs*8, leading to the synthesis of a non-functional DPD protein [29]. This resulted in a ~76% decrease in DPD activity in the two patients found to have this mutation; one of these patients died 28 days after chemotherapy, and the other experienced a grade 4 adverse event [29]. Interestingly, Saarenheimo et al. found four patients with a novel genomic exon 4 deletion, likely a result of an intronic mutation, within an unknown start site [17]. Similarly, this is predicted to cause a premature stop codon at p.Cys79Thrfs*8 [17]. This genomic exon 4 deletion was associated with an average 54% decrease in DPD activity in three patients where activity was determined. As such, none of these four patients received fluoropyrimidine chemotherapy [17]. This Finnish study reported 2.4% of patients (4/167) were carriers of this genomic exon 4 deletion [17], whereas we only found one carrier in 250 patients (0.2%) in our Canadian cohort. 

With emerging technology such as next-generation, whole-genome and exon sequencing becoming less costly and more accessible, the simultaneous discovery of new SNVs and CNVs is becoming more feasible. However, these methods are still costly, technical by nature, and require time-consuming data analysis. Alternatively, multiplex ligation-dependent probe amplification (MLPA) is a semi-quantitative method for the detection of SNV and CNV of up to 60 DNA sequences in a single reaction [39]. Probes are designed for specific DNA sequences, producing PCR amplicons of varying lengths which are quantified using capillary electrophoresis [39]. Using this method to determine *DPYD* CNV, probes can be designed for each exon to effectively determine deletions or duplications [17,18,29]. However, this method still requires the use of capillary electrophoresis, which is not a readily available technology in many laboratories. We have shown that the use of TaqMan assays to target a specific exon, in this case, exon 4, can easily detect CNV in *DPYD* and may be implemented more easily given the wide availability and use of TaqMan-based genotyping systems. 

In summary, a significant percentage of toxicity observed in patients during fluoropyrimidine chemotherapy which cannot be accounted for by the clinically relevant *DPYD* SNVs may be due to unrecognized *DPYD* CNVs. Accordingly, in addition to SNVs, the detection of CNVs in *DPYD*, such as exon 4, warrant consideration for inclusion for fluoropyrimidine pre-treatment *DPYD* deficiency testing, particularly in relevant populations.

## Figures and Tables

**Table 1:** Baseline Characteristics.

Characteristic	Case (N = 125)	Control (N = 125)
**Sex, N (%)**		
Female	63 (50)	63 (50)
Male	62 (50)	62 (50)
**Age in years, mean (SD)**	65.0 (10.4)	65.6 (9.9)
**Ethnicity, N (%) ^a^**		
Caucasian	124 (99)	114 (91)
African	0 (0)	2 (2)
Asian	1 (1)	1 (1)
Unknown	0 (0)	8 (6)
**Treatment characteristics**		
BSA (m^2^), mean (SD) ^b^	1.9 (0.2)	1.9 (0.3)
Initial Dose Intensity, mean (SD) ^c^	91 (14)	87 (14)
Average Dose Intensity, mean (SD)	81 (15)	84 (13)
Treatment Cycles, median (IQR) ^d^	6 (3–7)	6 (3–8)
**Regimen, N (%)**		
Capecitabine with radiation	14 (11)	13 (10)
Capecitabine monotherapy	35 (28)	35 (28)
Capecitabine with oxaliplatin	26 (21)	26 (21)
FOLFOX ^e^	31 (25)	31 (25)
FOLFIRI/FOLFIRINOX ^e^	13 (10)	14 (11)
5-FU with radiation	6 (5)	6 (5)

^a^ Ethnicity was self-declared by participants based upon their grandparents, in some cases the patients were unable to provide the information; ^b^ Body surface area; ^c^ Dose intensity reflects the percentage of ideal dose for each patient given their regimen and body surface area; ^d^ Number of treatment cycles attempted in each patient, some cycles were ended prematurely due to adverse events; ^e^ Includes patients with and without additional biologic therapy.

**Table 2:** Severe Fluoropyrimidine-related Adverse Events.

Category	No.
No. of Patients	125
No. of Adverse Events	157
**Gastrointestinal**	
Diarrhea	47
Colitis	11
Mucositis ^a^	6
Nausea/Vomiting ^b^	4
**Myelosuppression**	
Neutropenia	31
Febrile Neutropenia	11
Anemia	2
**HFS ^c^**	25
**Other ^d^**	15
**Death**	5

^a^ Includes oral mucositis and esophagitis; ^b^ Includes either nausea or vomiting; ^c^ Hand-foot syndrome (defined as palmar-plantar erythrodysesthesia syndrome); ^d^ Includes: hypokalemia, acute kidney injury, infection, fatigue and a maculopapular rash.

**Table 3:** Genomic deletions and duplications reported in *DPYD* resulting in phenotypic changes.

Gene Changes	Size	Effect on *DPYD*	Other Genes Affected	Phenotype	Ref.
1p21.3 deletion	10 kb	Deletion of exon 6	None	Autism, language delay	[19]
c.1340–3473_c.1525 + 10,154del1,3812	~13.8 kb	Deletion of exon 12	None	Seizures, aggressive attitude, developmental delay, muscular hypotonia, microcephaly, autistic-like behavior	[18]
c.1741_2058del	~122 kb	Deletion of exon 14–16	None	Amniotic infections, Respiratory insufficiency, developmental delay, facial and skeletal abnormalities, dysostosis multiplex	[18]
1p21.3 deletion	1.1 Mb	Whole *DPYD* deletion	*MIR137*	Severe language delay, aggressive behavior, autism, seizure	[19,20]
1p21.3 deletion	1.41 Mb	Whole *DPYD* deletion	*LOC729987, MIR137*	Mild intellectual disability, features of autism, tendency to overeat, remarkably shy and friendly, speech deficits, ocular problems	[21]
1p21.3 deletion	1.5 Mb	Whole *DPYD* deletion	*PTBP2*	Severe language delay, fine motor skill delay, autism, dysmorphic features	[19]
1p21.3 deletion	1.75 Mb	Whole *DPYD* deletion	*LOC729987, SNX7, LPPR5, MIR137*	Mild to moderate intellectual disability, features of autism, tendency to overeat, remarkably shy and friendly, ocular problem, facial structure abnormalities	[21]
1p21.3 deletion	2.45 Mb	Whole *DPYD* deletion	*LOC729987, PTBP2, MIR137, LOC101928241*	Mild intellectual disability, remarkable shy and friendly, aggressive outbursts	[21]
1p21.3p21.2 duplication	3.56 Mb	Whole *DPYD* duplication	*LOC101928241, PTBP2, MIR137 LOC729987, SNX7, LPPR5, LPPR4*	Intellectual disability, pervasive developmental disorder, febrile convulsions, psychomotor restlessness, hyperactivity, facial and skeletal abnormalities, clinodactyly	[22]
1p21.3 deletion	3.68 Mb	Whole *DPYD* duplication	*LOC101928241, PTBP2, MIR137, SNX7, LPPR5, LOC729987, LPPR4, PALMD, FRRS1, MIR548*	Intellectual disability	[22]
1p22.1p21.3 deletion	4.58 Mb	Whole *DPYD* deletion	*LOC101928241, PTBP2, MIR137, LOC729987*	Intellectual disability and obesity	[23]
1p21.3p21 deletion	5.43 Mb	Whole *DPYD* deletion	*PTBP2, MIR137, SNX7, LPPR5, LOC729987, LPPR4, LOC100129620*	Intellectual disability, autistic spectrum disorder	[23]
1p22.1p21.2 deletion	5.9 Mb	Whole *DPYD* deletion	*F3, LOC101928241, PTBP2, MIR137, SNX7, LPPR5, LOC729987, LPPR4, LOC100129620*	Neonatal hypotonia, psychomotor and speech delay, intellectual disability, obesity, hyperphagia, macrocephaly, ocular problems, clinodactyly	[23,24]
1p21.3p13.3 deletion	9.9 Mb	Partial *DPYD* deletion	*MIR137, SNX7, LPPR5, LOC729987, LPPR4, LOC100129620*	Delayed speech	[23]
1p21.3p13.3 deletion	11.19 Mb	Whole *DPYD* deletion	*LOC101928241, PTBP2, MIR137, SNX7, LPPR5, LOC729987, LPPR4, LOC100129620*	Intellectual disability and obesity	[23]
1p21.3p13.3 deletion	12 Mb	Whole *DPYD* deletion	*LOC101928241, PTBP2, MIR137, SNX7, LPPR5, LOC729987, LPPR4, LOC100129620, VCAM1, COL11A1, AMY2B, AMY2A, AMY1A*	Obesity, hyperphagia, psychomotor delay, speech delay, intellectual disability, macrocephaly precocious puberty	[23,24]
1p21.3p13.3 deletion	~14 Mb	Whole *DPYD* deletion	Multiple genes, including *WNT2B* and *NTNG1*	Intellectual disability, epilepsy, psychomotor and speech impairment, hypotonic and hypermobile, toe abnormalities, coloboma	[25]
1p21.3p13.3 deletion	14 Mb	Whole *DPYD* deletion	57 genes, including, *MIR137*, *PTBP2, SNX7*	Hypertonia and irritability at birth, hypotonia, areflexia, intellectual disability, facial and skeletal abnormalities, macrocephaly, epiphyseal dysplasia	[18]
1p22.3p13.3 deletion	Not reported	Whole *DPYD* deletion	Multiple genes	Intellectual disability, language delay, hypotonia, facial abnormalities, digitalized thumbs	[27]
1p22.3p13.3 deletion	Not reported	Whole *DPYD* deletion	Multiple genes	Intellectual disability, hearing loss, digitalized thumbs, toe abnormalities, facial abnormalities	[26]

## Data Availability

The data presented in this study are available on request from the corresponding author.
